# Effect of cumin (*Cuminum cyminum*) seed essential oil on biofilm formation and plasmid Integrity of *Klebsiella pneumoniae*

**DOI:** 10.4103/0973-1296.59967

**Published:** 2010-02-13

**Authors:** Safoura Derakhshan, Morteza Sattari, Mohsen Bigdeli

**Affiliations:** *Department of Bacteriology, School of Medical Sciences, Tarbiat Modares University, Tehran, Iran*; 1 *Department of Center of Agricultural Research, Tehran, Iran*

**Keywords:** Biofilm, *Cuminum cyminum*, essential oil, *Klebsiella pneumoniae*, plasmid

## Abstract

Seeds of the cumin plant *(Cuminum cyminum L.)* have been used since many years in Iranian traditional medicine. We assessed the effect of cumin seed essential oil on the biofilm-forming ability of *Klebsiella pneumoniae* strains and on the integrity of a native resistance plasmid DNA from *K. pneumoniae* isolates, treated with essential oil. Antibacterial coaction between the essential oil and selected antibiotic disks were determined for inhibiting *K. pneumoniae*. The essential oil of the cumin seeds was obtained by hydrodistillation in a Clavenger system. A simple method for the formation of biofilms on semiglass lamellas was established. The biofilms formed were observed by scanning electron microscopy (SEM). The effect of essential oil on plasmid integrity was studied through the induction of R-plasmid DNA degradation. The plasmid was incubated with essential oil, and agarose gel electrophoresis was performed. Disk diffusion assay was employed to determine the coaction. The essential oil decreased biofilm formation and enhanced the activity of the ciprofloxacin disk. The incubation of the R-plasmid DNA with essential oil could not induce plasmid DNA degradation. The results of this study suggest the potential use of cumin seed essential oil against *K. pneumoniae in vitro*, may contribute to the *in vivo* efficacy of this essential oil.

## INTRODUCTION

There is a continuing quest for safe and effective antimicrobial agents. This need has been heightened recently by the emergence of many antimicrobial-resistant organisms such as *Klebsiella pneumoniae*.[1] *K. pneumoniae* is an important Gram-negative pathogen, frequently associated with nosocomially acquired infections. It is involved in urinary tract infections, pneumonia, bacteremia, septicemia, and infections of surgical wounds. Whatever the infection site, the first stage of nosocomial infections due to *K. pneumoniae* consists of colonization in the patient's gastrointestinal tract.[2] Disruption of this ecosystem by antibiotics probably contributes to colonization by *K. pneumoniae*, as most of the strains involved are highly resistant to antibiotics.[1] Growth of *Klebsiella* strains as a biofilm mass occurs under a variety of environmental conditions.[3] Recent studies suggest that biofilm formation may be an important virulence factor for *K. pneumoniae*. Biofilm growth enhances resistance to antibiotic therapies, as well as host defense mechanisms.[4]

Cumin (*Cuminum cyminum* L.) is an aromatic plant included in the Apiaceae family and is used to flavor foods, added to fragrances, and used in medical preparations.[5] Its fruit, known as cumin seed, is yellow to brownish-gray in color and is elongated in shape with nine protuberances. Cumin possesses numerous medicinal properties. It is an aromatic herb and an astringent that benefits the digestive apparatus. It has been used in the treatment of mild digestive disorders as a carminative and eupeptic, as an astringent in broncopulmonary disorders, and as a cough remedy, as well as an analgesic.[6]

Our earlier report suggests that the essential oil of cumin seeds has a significant antibacterial activity against *K. pneumoniae in vitro*.[7] The identified essential oil components are given in Table 1. We have found that the growth of *K. pneumoniae* strains exposed to *C. cyminum* essential oil have resulted in cell elongation, repression of capsule expression, and inhibition of urease activity. In continuation of our previous work, the objective of the present study is to evaluate the biofilm inhibiting activity of cumin seed essential oil and the coaction of the essential oil with several antibiotics in inhibiting *K. pneumoniae.* The effect of the essential oil on the induction of resistance plasmid DNA degradation was also studied.

**Table 1:** Chemical composition of the *Cuminum cyminum* essential oil

Component	Scan number	KI^a^	Composition %
α-Thujene	606	930	0.2
α-Pinene	622	939	0.6
Sabinene	694	979	0.7
β-Pinene	705	982	10.3
Myrcene	728	993	0.8
α-Phellandrene	760	1008	0.4
ρ-Cymene	793	1023	7.2
β-Phellandrene	812	1031	0.7
γ-Terpinene	877	1058	19
ρ-Menth-3-en-7-al	1151	1152	5.1
Cumin aldehyde	1244	1179	25.2
ρ-Mentha-1,3-dien-7-al	1345	1206	13
ρ-Mentha-1,4-dien -7-al	1355	1209	16.6

a KI - Kovats Index on DB-I column, Reproduction size at column width

## MATERIALS AND METHODS

### Essential oil extraction

The seed parts of *C. cyminum* were collected from plants cultivated in the Center of Medicinal Plants Research, 25 km north of Tehran, Iran, and confirmed by the Center of Agricultural Research, Tehran, Iran.

The essential oil of the seeds was produced by the Clavenger apparatus, using the hydrodistillation method. The dried powdered seeds of cumin (50 g) were placed in a distillation apparatus with 1 L of distilled water and hydrodistilled for three hours. The oil was then removed and stored in sterile dark vials at 4°C until used.

### Test microorganisms

Six clinical isolates of *K. pneumoniae* were obtained from the Baqiyatullah Hospital (Tehran, Iran). *K. pneumoniae* ATCC13883 strain was purchased from Bou-Ali Reference Center (Tehran, Iran) and has been demonstrated to produce a good biofilm.[8] Clinical isolates were identified by standard methods for identification of Entrobacteriaceae.[9]

### Detection of biofilm inhibiting activity of essential oil

The biofilms of *K. pneumoniae* ATCC13883 and clinical isolates were grown on clean and sterile semiglass lamellas.[10] The semi glass lamellas were cut to identical diameters and sterilized by autoclave (121°C for 15 minutes) and placed in culture tubes containing growth medium and subinhibitory concentrations (sub-MIC) of essential oil, which were inoculated with each overnight culture (the MIC values were 0.8-3.5 μg/ml). A tube filled with growth medium alone was included as a negative control. The untreated cells were used as a positive control. Subsequently the tubes were incubated for 24 hours at 37°C. The quantitation of the biofilm remaining on the surfaces of the lamellas was performed by staining the bound cells, for two minutes, with a 1% aqueous solution of crystal violet as previously described.[11] After rinsing with distilled water, the bound dye was released from the stained cells by using 95% ethanol, and absorbance at 595 nm was determined. All assays were performed in duplicate and the results from the three experiments were reported.

To further investigate the ability of biofilm formation, imaging of the biofilm remaining on the surfaces of the lamellas was performed, using Scanning Electron Microscopy (SEM). All of the specimens were dried and mounted on aluminum stabs and sputter-coated with gold, with the help of an ion coater (SCDOOS; Bal-Tec, Balzers, Switzerland). Observation and photography were performed with a scanning electron microscope (XL30; Philips, Eindhoven, The Netherlands) operated at an acceleration voltage of 20 kV.[12]

### Effects of essential oil on the plasmid integrity[sugu1]

Plasmid DNA (R-plasmid) from clinical *K. pneumoniae* isolates was obtained by the alkaline lysis method as described by Sambrook *et al*.[13] LB-broth containing ampicillin (3.2 mg/ml) was inoculated with 10^6^ Cfu/ml of actively dividing bacterial cells. The cultures were incubated for 18-22 hours at 37°C. The plasmid DNA extractions were then performed. The RNA contaminants were digested by RNAse (100 μg/ml) treatment for 30 minutes at room temperature. For bacterial transformation, the competent bacteria were obtained as previously described.[13] The plasmids were transformed in an *E.coli* host strain, DH5α. The bacterial suspension (100 μl) was mixed with the plasmid DNA (0.5-1 μl) and incubated on ice for 30 minutes. After this, the tubes were placed on a water-bath at 42°C for one to two minutes following incubation on ice for three minutes, and 900 μl of pre-warmed LB (37°C) was then added to each of the tubes. The cultures were then incubated for one hour at 37°C and the aliquots were withdrawn and plated onto the LB-agar, containing ampicillin, in triplicate. The plates were incubated at 37°C for 24 hours.

To evaluate the role of essential oil in DNA breakage, plasmid DNA was dispensed in Eppendorf tubes (5 μl per tube) and incubated with 5 μl (35 μg/ml) of the essential oil. In all cases, the reaction mixtures were incubated at 37°C for 45 minutes. After treatment, the DNA was electrophoresed in 0.8% agarose gel. Aliquots of each sample (6 μl) were mixed with 2 μl of 6X concentrated loading buffer (0.25% xylene cyanol; 0.25% bromophenol blue; 30% glycerol in water), applied in a horizontal gel in Tris acetate - EDTA buffer (1 × TAE buffer, pH 8.0), and performed at 60 V. After electrophoresis, the gel was stained with ethidium bromide (10 mg/ml) and the DNA bands were visualized by fluorescence in an ultraviolet (UV) DNA transilluminator system (Germe-tec, Sao Paulo, Brazil) at 254 nm. Untreated R-plasmid DNA was used as a control.

Determ ining of the coaction of the essential oil with selected antibiotic disks

The disk diffusion assay described by Bauer *et al.*,[14] was employed to determine the coaction between the antimicrobial agents. Used antibiotic disks (Padtan Teb, Tehran, Iran) were included: Ciprofloxacin (5 μg), amoxicillin (25 μg), nalidixic acid (30 μg), trimethoprim-sulfamethoxazole (SXT), ceftazidime (30 μg), cefixime (5 μg), and tetracycline (30 μg).

Briefly, all bacteria were grown to a logarithmic phase in the broth media. A logarithmic phase culture of 0.1 ml volume was spread over the surface of the Mueller-Hinton agar (Hispanlab, Madrid, Spain) plates containing subminimum inhibitory concentrations (sub-MICs) of the essential oil and antibiotic disks were placed on the surface of agar plates. The plates were then incubated for 18-22 hours at 37°C and examined for zones of inhibition. Plates with no essential oil supplement were used as the control. If the zone remained unchanged there was no coaction. However, if there was an increase in the zone size, it could be concluded that there was synergy. All tests were performed in duplicate.

## RESULTS

### Effect of essential oil on the *K. pneumoniae* biofilm formation

**Figure 1:**
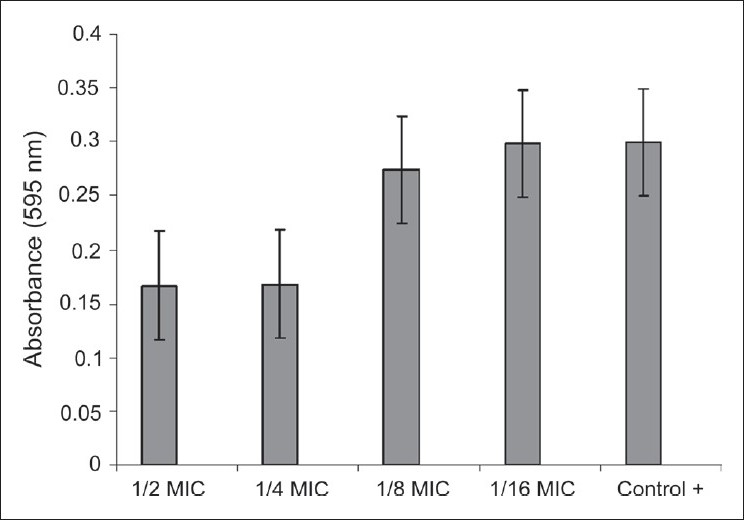
Biofilm formation on the surface of lamellas in broth media containing essential oil dilution by *Klebsiella pneumoniae* ATCC 13883. The bars indicate means ± standard errors of the means (error bars) from three experiments. (Reproduction size at column width)

The changes of *K. pneumoniae* biofilms brought about by treatment with various concentrations of essential oil are shown in Figure 1. Strains that were exposed to sub-MIC levels of essential oil exhibited a reduction of 50% or more in the OD_595_ reading compared to the control. These results showed that these strains exhibited an impaired ability to form a biofilm compared to the control.

**Figure 2:**
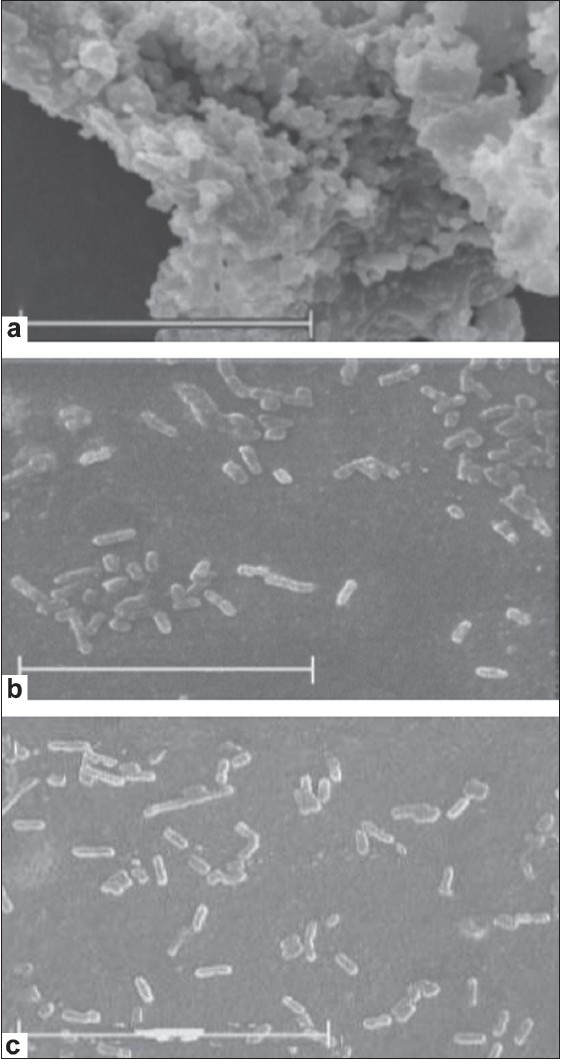
SEM images of *Klebsiella pneumoniae* ATCC 13883 biofilms exposed to sub-MICs of essential oil of *Cuminum cyminum* seeds. Biofilms of *K. pneumoniae* formed by 24-hour incubation, in the presence of essential oil. Preparation and observation under the SEM were carried out as described in the text. The bar represents 20 μm. (Reproduction size at column width) a: Control. A biofilm formed in the medium without essential oil. b: Formed in the presence of 1/4 × MIC of essential oil (0.2-0.87 μg/ml). c: Formed in the presence of 1/2 × MIC of essential oil (0.4-1.75 μg/ml)

The cell density and cell distribution in the biofilms of *K. pneumoniae* were clearly defined by SEM images [Figure 2]. After 24 hours, *C. cyminum* reduced the extent of the biofilm formation, as a function of increasing essential oil concentration. In the control biofilm, the cells formed a dense aggregate [Figure 2a], whereas, in the sub-MIC concentration of essential oil, the cells were scattered [Figure 2b and c]. Greater changes in the biofilm formation extent were obtained with levels of 1/2 × MIC (0.4-1.75 μg/ml) than in 1/4 × MIC (0.2-0.87 μg/ml).

### Effect of the essential oil on plasmid integrity

**Figure 3:**
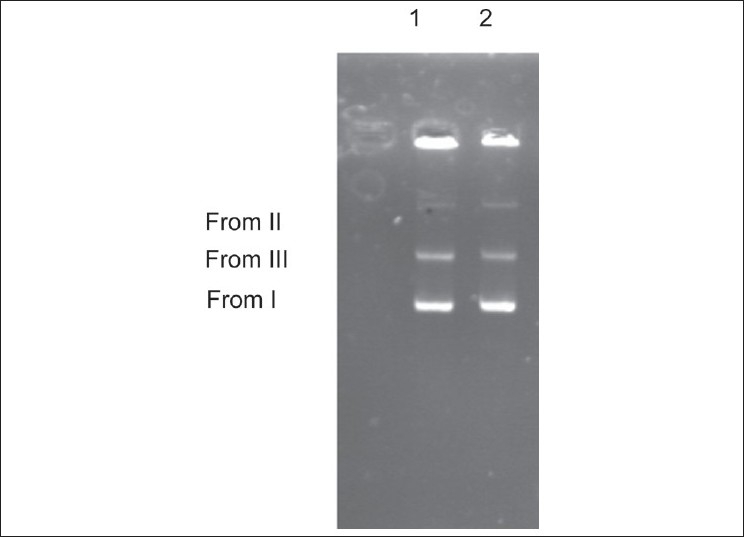
A percentage of 0.8% agarose gel electrophoresis of the plasmid (resistance plasmid) treated with essential oil. Aliquots of R-plasmid DNA (5 μl) were incubated with 5 μl (35 μg/ml) of the essential oil for 45 minutes at 37°C. After incubation, the reaction mixtures were submitted to agarose gel electrophoresis. Lane 1: Plasmid extracted from isolate 1 plus essential oil. Lane 2: Plasmid extracted from isolate 2 plus essential oil. I = supercoiled form (SC); II = open circle form (OC), and III = linear form (L). (Reproduction size at column width)

In this study, we investigated the effect of the essential oil of *C. cyminum* on plasmid DNA integrity (R-plasmid). Of the six clinical isolates examined, two isolates were found to carry the R-plasmid (isolates 1 and 2). Figure 3 shows the results obtained from a typical agarose gel electrophoresis of R-plasmid DNA, which was incubated with 5 μl of essential oil (35 μg/ml). DNA derived from the reaction mixtures showed three bands on agarose gel electrophoresis; form I: The faster moving prominent band corresponded to the native supercoiled circular DNA (SC DNA), form II: Open circle (OC) resulting from single strand breaks, and form III: Linear (L) resulting from double strand breaks. The results showed that essential oil, up to 35 μg/ml, did not cause any degradation of plasmid DNA [Figure 3, lanes 1 and 2]. The control reaction has no damaging effect on plasmid DNA.

### Coaction between essential oil and antibiotic disks

The disk diffusion assay as described in the ‘Materials and Methods’ was used to detect any coaction that existed between the essential oil and several antibiotic disks. Of the antibiotics investigated, the greatest positive coaction was seen between essential oil and ciprofloxacin (data not shown). Surprisingly, antagonistic coaction was seen between the oil and the trimethoprim-sulfamethoxazole disk. There was no apparent difference in the diameter of inhibition zones of other antibiotic disks as compared to the control.

## DISCUSSION

Emergence of multidrug resistance bacteria is threatening world population. Thus, the search continues for new antimicrobials from other sources, including plants. Medicinal plants are considered as potential sources of new chemotherapeutic drugs because of their diverse phytochemicals and little or no toxic effect.[15]

*K. pneumoniae* produced copious amounts of an acidic polysaccharide capsules, which allowed it to adhere to epithelial cells and form biofilms on abiotic surfaces.[2] Recent studies have suggested that biofilm formation may be an important virulence factor for *K. pneumoniae*.[4] In our investigation, the results showed that strains exposed to the sub-MIC concentration of essential oil exhibited a reduction of two-fold or more in the OD_595_ reading compared to the control. SEM images showed that these strains had an impaired ability to form a biofilm, and the cells of the surface were scattered, compared to the control biofilm. Our report clearly suggested that the essential oil of cumin seeds reduced the expression of the *K. pneumoniae* capsular layer.[7] This could be an important factor that resulted in the reduction of biofilm formation. Also, it was suggested that essential oils acted with the help of their lipophilic fraction reacting with the lipid parts of the cell membranes;[16] investigation showed that damaging of the microbial membrane structures could influence biofilm formation.[17] Therefore, in the present study, damaging of the microbial membrane structures could have influenced biofilm formation.

We found that *C. cyminum* essential oil could not induce R-plasmid DNA degradation. Considering the molecular characteristics of the aldehydes present in a good amount in our oil,[7] we can hypothesize that these compounds may have contributed to the observed activity of the tested essential oil.[18]

The mechanism by which the cumin seed essential oil acts, to enhance the activity of ciprofloxacin against *K. pneumoniae*, as indicated by an increased inhibition zone diameter, is not known; but some cell wall damage or alteration in the outer membrane proteins may be caused by the cumin seed essential oil, which sensitizes cells to ciprofloxacin as well as resists cells to trimethoprim-sulfamethoxazole.[19,20] The enhancement of antibacterial efficacy of ciprofloxacin by the essential oil is significant. Cumin seed essential oil, in combination with antibiotics, may be used in medicines. However, the ability of this essential oil to decrease the activity of trimethoprim-sulfamethoxazole, limits its usefulness in some medicinal applications.

## CONCLUSION

Results of this study suggest that the essential oil of cumin seed may be useful either alone or when combined with antimicrobial agents, to treat bacterial infections. The antibacterial properties of cumin essential oil are mostly attributable to the cumin aldehyde. Further studies are necessary to evaluate the possible toxicity of this essential oil and its application in the medicinal system, before any claims can be made.
